# Mini Review Immunological Consequences of Immunization With COVID-19 mRNA Vaccines: Preliminary Results

**DOI:** 10.3389/fimmu.2021.657711

**Published:** 2021-03-12

**Authors:** Andrea Lombardi, Giorgio Bozzi, Riccardo Ungaro, Simone Villa, Valeria Castelli, Davide Mangioni, Antonio Muscatello, Andrea Gori, Alessandra Bandera

**Affiliations:** ^1^Infectious Diseases Unit, Foundation Istituto di Ricovero e Cura a Carattere Scientifico (IRCCS) Ca' Granda Ospedale Maggiore Policlinico, Milan, Italy; ^2^Department of Pathophysiology and Transplantation, University of Milano, Milan, Italy; ^3^Center for Multidisciplinary Research in Health Science (MACH), University of Milano, Milan, Italy

**Keywords:** COVID-19, SARS-CoV-2, RNA vaccine, CTL, antibodies, neutralization test

## Abstract

**Background:** BNT162b2 and mRNA-1273 are the two recently approved mRNA-based vaccines against COVID-19 which has shown excellent safety and efficacy. Preliminary data about specific and neutralizing antibodies is available covering the first 100 days after vaccination.

**Methods:** We reviewed all the publications regarding the immunologic consequences of BNT162b2 and mRNA-1273 vaccination. A summary of specific antibodies concentration and neutralizing antibodies titers elicited by each vaccine is provided.

**Results:** BNT162b2 and mRNA-1273 displayed a reassuring safety and efficacy profile, with the latter above 94%. They can elicit specific antibodies titers and neutralizing antibodies concentrations that are far superior from those observed among COVID-19 human convalescent serum, across a wide span of age, for at least 100 days after vaccination. Moreover, the vaccine-induced T cellular response is oriented toward a T_H_1 response and no evidence of vaccine-enhanced disease have been reported.

**Discussion:** BNT162b2 and mRNA-1273 can elicit specific antibodies titers and neutralizing antibodies concentrations above those observed among COVID-19 human convalescent serum in the first 100 days after vaccination. Data about vaccine efficacy in those with previous COVID-19 or immunocompromised is still limited.

## Introduction

Severe Acute Respiratory Syndrome Coronavirus 2 (SARS-CoV-2) is the beta-coronavirus responsible for the disease called Coronavirus Disease 2019 (COVID-19). Currently, SARS-CoV-2 has infected over 75 million people and has caused 1.6 million deaths (1). To date, no specific therapies are available. Mass prophylactic vaccination is probably the only viable path to cost-effectively curb out the pandemic.

The ability of humoral and cellular immunity to protect from SARS-CoV-2 has been clearly demonstrated. In rhesus macaques the adoptive transfer of purified IgG from convalescent to naïve animals protected the latter from SARS-CoV-2 challenge in a dose dependent fashion. The depletion of T CD8^+^ cell from convalescent macaques partially abrogated the protective efficacy of natural immunity against SARS-CoV-2 re-challenge, suggesting the importance of cellular immunity (2). In humans, a study conducted on healthcare professionals, showed how the presence of anti-spike or anti-nucleocapsid antibodies was associated with a significant reduction in the risk of reinfection throughout an observation period of over 6 months (3).

Less clear is the duration of the protection provided by a previous infection. Most long-term studies performed in patients with either Severe Acute Respiratory Syndrome Coronavirus (SARS-CoV) or Middle East respiratory syndrome coronavirus (MERS-CoV) infection, found that IgG titers waned over time (typically remaining detectable up to at least a year), while others found detectable levels of IgG up to 3 years after symptom onset (4). Despite this apparent decline of specific IgG, long-lasting memory T cells reactive to the nucleocapsid protein of SARS-CoV were found in individual 17 years after the infection, suggesting that beta-coronaviruses infection can induce long-lasting T cell immunity (5). In COVID-19, kinetics of the neutralizing antibody response is typical of an acute viral infection, with declining neutralizing antibody titers observed after an initial peak, which depends on disease severity (6). Recently, it has been estimated that it takes at least 372, 416, and 133 days for plaque reduction neutralization assays with 50% end-point (PRNT50) titers to drop to the detection limit for severe, mild and asymptomatic patients, respectively (7). High expectations are therefore placed on vaccines, for both the immunization of naïve population and eventually the boosting of already achieved immunity among previously infected patients.

In this review, we describe the immunologic effects of BNT162b2 or mRNA-1273 vaccines, updating with the results of phase III trials of mRNA vaccines the superb paper of Krammer (8). A summary of specific antibodies concentration (Table 1) and neutralizing antibodies (NA) titers (Table 2) elicited by each vaccine is provided, with stratification per age of the participants, dose administered, and relative ratio compared to values detected in human COVID-19 convalescent serum (HCS). We also discuss the role of these vaccines among special populations and the evidence available on the feared vaccine-enhanced immune response.

**Table 1:** Vaccine-induced IgG concentrations from available studies of BNT162b1, BNT162b2 and mRNA-1273 vaccines.

**Vaccine**	**Dose (μg)**	**Age group (years)**	**Participants**	**Days after vaccine first dose**	**Titer IgG vaccinated**	**Ratio**	**Titer IgG HCS**	**Reference**
BNT162b1^*^	10	19–54	12	7	0.9	0.00	602	(9)
				21	534	0.89		
				28	4,813	8.00		
				35	5,880	9.77		
	30	19–54	12	7	0.8	0.00		
				21	1,536	2.55		
				28	27,872	46.30		
				35	16,166	26.85		
	100	19–54	12	7	1.2	0.00		
				21	1,778	2.95		
				28	1,260	2.09		
BNT162b1^†^	10	18–55	12	21	294	0.47	631	(10)
				28	3,868	6.13		
				35	5,120	8.11		
	20	18–55	12	21	204	0.32		
				28	3,243	5.14		
				35	7,480	11.85		
	30	18–55	12	21	853	1.35		
				28	23,516	37.27		
				35	13,940	22.09		
	10	65–85	12	21	36	0.06		
				28	980	1.55		
				35	1,527	2.42		
	20	65–85	12	21	125	0.20		
				28	10,623	16.84		
				35	6,399	10.14		
	30	65–85	12	21	86	0.14		
				28	6,580	10.43		
				35	4,798	7.60		
BNT162b2^†^	10	18–55	12	21	612	0.97		
				28	5,782	9.16		
				35	4,717	7.48		
	20	18–55	12	21	547	0.87		
				28	12,464	19.75		
				35	7,367	11.68		
	30	18–55	12	21	1,265	2.00		
				28	9,136	14.48		
				35	8,147	12.91		
	10	65–85	12	21	172	0.27		
				28	3,203	5.08		
				35	3,560	5.64		
	20	65–85	12	21	81	0.13		
				28	3,056	4.84		
				35	2,656	4.21		
	30	65–85	12	21	329	0.52		
				28	7,985	12.65		
				35	6,014	9.53		
BNT162b1^*^	1	20–56	12	8	3	0.00	602	(11)
				22	265	0.44		
				29	2,015	3.35		
				43	3,920	6.51		
	10	20–56	12	8	1	0.00		
				22	826	1.37		
				29	9,107	15.13		
				43	6,707	11.14		
	30	20–56	12	8	2	0.00		
				22	1,273	2.11		
				29	17,051	28.32		
				43	12,431	20.65		
	50	20–56	12	8	1	0.00		
				22	1,672	2.78		
				29	25,006	41.54		
				43	18,289	30.38		
	60^‡^	20–56	12	8	1	0.00		
				22	909	1.51		
				29	1,058	1.76		
				43	755	1.25		
mRNA-1273^•^	25	18–55	15	1	0.5	0.00	14,196	(12)
				15	5,674	0.40		
				29	8,304	0.58		
				36	94,998	6.69		
				43	91,081	6.42		
				57	77,904	5.49		
	100	18–55	15	1	1.3	0.00		
				15	19,068	1.34		
				29	20,525	1.45		
				36	213,076	15.01		
				43	221,956	15.64		
				57	147,332	10.38		
	250	18–55	15	1	1.4	0.00		
				15	30,641	2.16		
				29	38,448	2.71		
				36	315,723	22.24		
				43	254,374	17.92		
				57	215,140	15.15		
mRNA-1273^♢^	25	56–70	10	1	189	0.00	138,901	(13, 14)
				15	10,509	0.08		
				29	17,684	0.13		
				36	313,720	2.26		
				43	476,136	3.43		
				57	323,945	2.33		
	25	≧71	10	1	111	0.00		
				15	14,837	0.11		
				29	57,986	0.42		
				36	460,094	3.31		
				43	303,630	2.19		
				57	1,128,391	8.12		
	100	56–70	10	1	655	0.00		
				15	55,532	0.40		
				29	115,831	0.83		
				36	5,033,017	36.23		
				43	1,305,996	9.40		
				57	1,183,066	8.52		
			9	119	151,761	1.09		
	100	≧71	10	1	953	0.01		
				15	84,383	0.61		
				29	203,365	1.46		
				36	2,636,979	18.98		
				43	8,091,439	58.25		
				57	3,638,522	26.20		
				119	157,946	1.14		
	100	18–55	15	1	131	0.00		
				15	86,291	0.62		
				29	109,209	0.79		
				36	781,399	5.63		
				43	811,119	5.84		
				57	782,719	5.64		
				119	235,228	1.70		

*The ratio between the titer induced by the vaccine and the titer identified in the COVID-19 convalescent control population of the study is presented in the homonymous column*.

* *GMC RBD-binding IgG, Luminex assay (U mL-1)*;

† *GMC S1-binding IgG, Luminex assay (U mL-1)*;

‡ *not boosted*;

• *IgG ELISA S-2P area under the curve IgG Williams mean*;

♢ *GMC ELISA anti-S-2P; HCS, Human coronavirus disease 2019 (Covid-19) or SARS-CoV-2 infection convalescent serum; GMC geometric mean concentration*.

**Table 2:** Vaccine-induced virus neutralization titers from available studies of BNT162b1, BNT162b2 and mRNA-1273 vaccines.

**Vaccine**	**Dose**	**Age group**	**Participants**	**Days after vaccine first dose**	**GMT neutralizing Abs**	**Ratio**	**GMT neutralizing Abs HCS**	**Reference**
BNT162b1^*^	10	19–54	12	7	10	0.11	94	(9)
				21	13	0.14		
				28	168	1.79		
				35	180	1.91		
	30	19–54	12	7	10	0.11		
				21	29	0.31		
				28	267	2.84		
				35	437	4.65		
	100	19–54	12	7	10	0.11		
				21	33	0.35		
BNT162b1^*^	10	18–55	12	21	13	0.14	94	(10)
				28	168	1.79		
				35	180	1.91		
	20	18–55	12	21	13	0.14		
				28	160	1.70		
				35	203	2.16		
	30	18–55	12	21	29	0.31		
				28	267	2.84		
				35	437	4.65		
	10	65–85	12	21	11	0.12		
				28	20	0.21		
				35	33	0.35		
	20	65–85	12	21	10	0.11		
				28	179	1.90		
				35	105	1.12		
	30	65–85	12	21	12	0.13		
				28	101	1.07		
				35	105	1.12		
BNT162b2^*^	10	18–55	12	21	16	0.17		
				28	157	1.67		
				35	97	1.03		
	20	18–55	12	21	19	0.20		
				28	163	1.73		
				35	292	3.11		
	30	18–55	12	21	14	0.15		
				28	361	3.84		
				35	163	1.73		
	10	65–85	12	21	10	0.11		
				28	79	0.84		
				35	111	1.18		
	20	65–85	12	21	10	0.11		
				28	84	0.89		
				35	81	0.86		
	30	65–85	12	21	12	0.13		
				28	149	1.59		
				35	206	2.19		
BNT162b1^*^	1	20–56	12	8	10	0.11	94	(11)
				22	12	0.13		
				29	36	0.38		
				43	62	0.66		
	10	20–56	12	8	10	0.11		
				22	21	0.22		
				29	158	1.68		
				43	126	1.34		
	30	20–56	12	8	10	0.11		
				22	28	0.30		
				29	308	3.28		
				43	157	1.67		
	50	20–56	12	8	10	0.11		
				22	32	0.34		
				29	578	6.15		
				43	333	3.54		
	60	20–56	12	8	10	0.11		
				22	19	0.20		
				29	20	0.21		
				43	17	0.18		
mRNA-1273^†^	25	18–55	15	1	10	0.23	43.1	(12)
				15	10.9	0.25		
				29	10	0.23		
				36	53.1	1.23		
				43	59.9	1.39		
				57	39.1	0.91		
	100	18–55	15	1	10	0.23		
				15	12.8	0.30		
				29	10.2	0.24		
				36	120.7	2.80		
				43	153.8	3.57		
				57	110.3	2.56		
	250	18–55	15	1	10	0.23		
				15	13.4	0.31		
				29	12.4	0.29		
				36	158	3.67		
				43	140.9	3.27		
				57	120.8	2.80		
mRNA-1273^‡^	25	56–70	10	1	–	–	41	(13, 14)
				15	–	–		
				29	–	–		
				36	79	1.93		
				43	116	2.83		
				57	92	2.24		
	25	≥71	10	1	–	–		
				15	–	–		
				29	–	–		
				36	121	2.95		
				43	112	2.73		
				57	86	2.10		
	100	56–70	10	1	–	–		
				15	11	0.27		
				29	–	–		
				36	289	7.05		
				43	402	9.80		
				57	324	7.90		
			9	119	167	4.07		
	100	≥71	10	1	–	–		
				15	26	0.63		
				29	16	0.39		
				36	310	7.56		
				43	317	7.73		
				57	242	5.90		
				119	109	2.66		
	100	18–55	15	1	–	–		
				15	24	0.59		
				29	18	0.44		
				36	263	6.41		
				43	360	8.78		
				57	267	6.51		
				119	182	4.43		

*The ratio between the titer induced by the vaccine and the titer identified in the COVID-19 convalescent control population of the study is presented in the homonymous column*.

* *50% SARS-CoV-2-neutralizing GMTs*;

† *Pseudovirus neutralization assay ID80 geometric mean*;

‡ *Pseudotyped lentivirus reporter neutralization assay ID50; GMT, geometric mean titer; HCS, Human coronavirus disease 2019 (Covid-19) or SARS-CoV-2 infection convalescent serum*.

## The Innovation of RNA Vaccines

The first experiments on RNA inoculation in animal models date back to the 1990s (15). However, before COVID-19, no RNA-based vaccine has never been approved nor remarkable progresses have been made in their use in clinical trials.

The concept behind the use of RNA–either self-replicating or not–, namely a messenger RNA (mRNA), is simple: inoculate a molecule (i.e., the RNA) carrying the genetic information coding for the antigen, which will be produced via the ribosomal machineries of the host cells, rather than injecting the antigen itself to trigger the immune response.

Since the start of the COVID-19 pandemic, besides RNA vaccines, several “next-generation” vaccine platforms have been employed, like: viral vectors, DNA vaccine, and antigen-presenting cells (16). To date, only two non-self-replicating mRNA vaccine against COVID-19 have been conditionally approved by independent regulatory agencies.

## BNT162b2 (Pfizer/BioNTech)

BNT162b2 was the first vaccine to be granted a conditional marketing approval by the European Medicines Agency (EMA) for the prevention of COVID-19 (17). BNT162b2 is administered intramuscularly after dilution as a cycle of 2 doses (0.3 mL each, containing 30 μg of COVID-19 mRNA vaccine embedded in lipid nanoparticles), 21 days apart (18). BNT162b2 consists of a nucleoside-modified RNA, contained within a lipid nanoparticle, which encodes the SARS-CoV-2 full-length spike, modified by two proline mutations to lock it in the prefusion conformation (10). In the initial phases of drug development, a similar product was concomitantly assessed, BNT162b1, which encodes the SARS-CoV-2 receptor–binding domain (RBD) (10). BNT162b1 is not anchored on the membrane like BNT162b2 but instead secreted.

Preclinical data investigated BNT162b2 immunogenicity and anti-viral properties in mice and non-human primates. Immunization of mice with a single dose of BNT162b2 induced dose level-dependent increases in pseudovirus neutralization titers. Prime-boost vaccination of rhesus macaques elicited authentic SARS-CoV-2 neutralizing geometric mean titers 10.2 to 18.0 times that from SARS-CoV-2 HCS. From the cellular point of view, BNT162b2 generated strong T_H_1 type CD4^+^ and IFNγ^+^ CD8^+^ T-cell responses in both mice and rhesus macaques. Finally, the BNT162b2 vaccine candidate fully protected the lungs of immunized rhesus macaques from infectious SARS-CoV-2 challenge (19).

In a Phase I/II study (NCT04368728) conducted among 45 adult participants with 18–55 years of age, BNT162b1 was administered at doses of 10 or 30 μg in two administrations 21 days apart (9). Twelve participants received a single 100 μg dose at day 1 and other 9 participants received placebo. Robust immunogenicity was documented with all vaccine doses. By 21 days after the first dose, geometric mean concentrations (GMCs) of RBD-binding IgG ranged from 534 to 1,778 U·ml^−1^ compared to a value of 602 U·ml^−1^ observed in a panel of 38 HCS drawn at least 14 days after a PCR-confirmed diagnosis. Seven days after administration of the second dose, with 10 or 30 μg of BNT162b1, the GMC of RBD-binding IgG was ~8.0–50 times higher the GMC of HCS and remained far superior also at the last evaluation performed, 14 days after the second dose. This higher vaccine-induced GMC of RBD-binding IgG compared to GMC of HCS can be attributed, in part, to epitope-binding antibodies that are exposed to the RBD immunogen expressed after vaccination but are inaccessible to antibodies in RBDs that are embedded in the tips of SARS-CoV-2 virions during the infection. Regarding the geometric mean titer (GMT) of NA this, after the second dose of 10 and 30 μg was, respectively, 1.8 and 2.8 times the GMT of the serum panel from HCS. At day 35 (i.e.,14 days after the second dose), the GMT of NA had increased, up to 1.9 and 4.6-fold the GMT of the HCS, for the 10 and 30 μg doses, respectively. This is consistent with the antibody affinity maturation process.

Comparable results were obtained in a second placebo-controlled, observer-blinded, dose-escalation, phase 1 trial (NCT04368728), which compared the safety and immunogenicity of BNT162b1 and BNT162b2 among 195 adults aged 18–55 or 65–85 years (10). In all groups but one, participants received two doses (10, 20, or 30 μg), with a 21-day interval between doses; in one group (100 μg of BNT162b1), participants received one dose. The 50% neutralizing GMTs for the two vaccines, on day 28 or 35, was between 1.7 and 4.6 times the GMT of the HCS among participants aged 18–55 years and 1.1–2.2 times the GMT of HCS among subjects aged 65–85. Immunogenicity was reduced in those older than 65 years of age compared to younger participants but remained relevant. Overall, BNT162b2 was associated with a lower incidence and severity of systemic reactions than BNT162b1, particularly in older adults, and therefore was chosen for advancing to phase II/III studies.

Clearly, the mere ability to generate specific antibodies for the epitope encoded by the vaccine alone is not indicative of efficacy in protecting against SARS-CoV-2 infection. Therefore, the efficacy and safety of BNT162b2 were verified in a large multinational, placebo-controlled, observer-blinded, efficacy trial (NCT04368728) which involved 43,548 participants (20). Regarding efficacy, in participants without evidence of previous infection, eight cases of COVID-19 were recorded starting 7 days after the administration of the second dose among vaccinated and 162 cases among those who had received the placebo. Overall, BNT162b2 showed 95% efficacy [95% credible interval (CI), 90.3 to 97.6] in preventing COVID-19. It is interesting to note that between first and second dose, 39 cases of COVID-19 were observed in the BNT162b2 arm and 82 cases in the placebo one, with an efficacy of 52% during this interval. This indicates early protection by the vaccine, which appears from 12 days after the first dose. Although a previous SARS-CoV-2 infection represented an exclusion criterion from the study, some subjects with previous COVID-19 were enrolled (3,614/40,137; 9%). Including also these participants in the analysis, from 7 days after the second dose, nine cases of COVID-19 were observed among vaccine recipients and 169 among placebo recipients, achieving an efficacy of 94.6% (95% CI, 89.9–97.3).

Some more thorough data on the impact of vaccination with BNT162b1 (NCT04380701) on the humoral and adaptive cellular immune response have recently been made available and confirmed the data from the phase I/II studies (11). In this study, 60 participants were vaccinated with BNT162b1, 12 for each of the dose level groups (1, 10, 30, and 50 μg) received the first dose on day 1 and a booster dose on day 22 and 12 participants received only a 60 μg prime dose on day 1. The induction of high titers of anti-RBD antibodies was confirmed. At day 43 (21 days after dose II administration), the GMC of anti-RBD antibodies ranged from 3,920 to 18,289 U·ml^−1^ among vaccinated, compared with a GMC of 602 U ml^−1^ in HCS. Among participants who had received only the first dose, the GMC of anti-RBD antibodies was 755 U·ml^−1^, indicating the need of a booster to achieve a significant titer. Also, the production of NA was confirmed. The GMT of NA, 7 days after the second dose, was 36 (1 μg dose), 158 (10 μg dose), 308 (30 μg dose), and 578 (50 μg dose), compared to 94 for HCS. It is interesting to note that at day 43, both the GMT of NA and the GMC of anti-RBD antibodies were decreasing compared to previous detections. To verify the efficacy of the vaccine also against viral variants with alterations in the nucleotide sequence encoding the viral spike, sera from vaccinated participants 7 days after the booster dose were tested against a panel of 16 viral variants, showing in all cases a high neutralizing capacity. Moreover, a recent publication assessed the GMT of NA against the United Kingdom (UK) and South African (SA) variants of SARS-CoV-2 in individuals immunized with BNT162b2. The virus was engineered to contain the following mutations in the spike sequence: N501Y from UK and SA; 69/70-deletion + N501Y + D614G from UK; and E484K + N501Y + D614G from SA. The sera, drawn 2 or 4 weeks after immunization, showed equivalent neutralization titers between the wild-type and mutant viruses. Apparently, the neutralization GMT of the serum panel against the virus with three mutations from the SA variant (E484K + N501Y + D614G) was slightly lower than the neutralization GMTs against the N501Y virus or the virus with three mutations from the UK variant (Δ69/70 + N501Y + D614G) (21). A rough assessment of the adaptive cellular response was also provided: 95.2% of the subjects mounted an RBD-specific CD4^+^ T cell response and the intensity of this response was directly correlated with the GMC of anti-RBD antibodies and the GMT of NA. A specific CD8^+^ T response was detected in 76.2% of the vaccinated, with a direct correlation with the CD4^+^ T response but not with the GMT of NA. Both CD4^+^ and CD8^+^ RBD-specific T lymphocytes secreted IFNγ+ and IL-2. Finally, intracellular cytokine analysis identified a functional and pro-inflammatory response in both CD4^+^ and CD8^+^ T lymphocytes, with a T_H_1 orientation consisting in the production of TNF, IL-1β and IL-12p70, but neither IL-4 nor IL-5 (11).

## mRNA-1273 (Moderna/NIH)

The second vaccine employing mRNA approved by the EMA, is mRNA-1273 (22). mRNA-1273 is a lipid nanoparticle–encapsulated, nucleoside-modified messenger RNA-based vaccine that encodes the SARS-CoV-2 spike glycoprotein stabilized in its prefusion conformation (12). mRNA-1273 has been licensed for active immunization to prevent COVID-19 in individuals aged 18 years and older and it is administered as a course of 2 doses (0.5 mL each, containing 100 μg of messenger RNA embedded in SM-102 lipid nanoparticles), 28 days apart (23).

Preliminary results of immunogenicity were obtained in mouse model where two intramuscular injections with 0.01, 0.1, or 1 μg of mRNA-1273, 3 weeks apart, induced dose-dependent specific spike-binding antibodies after prime and boost in all mouse strains. Potent pseudovirus-neutralizing activity was elicited by 1 μg mRNA-1273, reaching reciprocal half-maximal inhibitory concentration (IC50) GMTs of 819 (BALB/cJ), 89 (C57BL/6J), and 1,115 (B6C3F1/J). Of note, neutralizing activity was conserved also against pseudoviruses expressing spike protein with the D614G substitution (24). Interesting data were provided about the balance of T_H_1 and T_H_2 cells, the vaccination induced both IgG2a and IgG1 subclasses, indicating a equilibrated T_H_1–T_H_2 response. After restimulation with a peptide pools covering the entire spike protein, splenocytes from immunized mice secreted more IFN-γ than IL-4, IL-5, or IL-13, suggesting a T_H_1-dominant response. This T_H_1-oriented response was confirmed also by the evaluation of intracellular cytokines in CD4^+^ T cells re-stimulated with spike peptide pools 7 weeks after immunization. Overall, immunization with mRNA-1273 elicits balanced T_H_1 and T_H_2 responses in mice, with a dose-dependent protection from viral replication and immunopathology in nose and lung of mice (24).

These results were confirmed also in nonhuman primate models which were immunized with 10 or 100 μg of mRNA-1273. The vaccination induced antibody levels exceeding those in HCS, with live-virus reciprocal 50% inhibitory dilution GMT of 501 in the 10-μg dose group and 3,481 in the 100-μg dose group. Vaccination induced T_H_1–biased (interferon-γ, interleukin-2, or tumor necrosis factor α) CD4^+^ T-cell responses and low or undetectable T_H_2 (interleukin-4 or 13) or CD8^+^ T-cell responses. Viral replication was not detectable in bronchoalveolar lavage fluid by day 2 after challenge in seven of eight animals in both vaccinated groups. No viral replication was detectable in the nose of any of the eight animals in the 100 μg dose group by day 2 after challenge, and limited inflammation or detectable viral genome or antigen was noted in lungs of animals in either vaccine group (25).

In two publications of phase I study (NCT04283461), mRNA-1273 was administered twice at doses of 25, 100, and 250 μg 28 days apart in 45 healthy adults, 18–55 years of age (12), and in 40 healthy adults over 56 years of age at doses of 25 and 100 μg (13). On day 57 from the prime dose of 100 μg, the GMT of specific anti-RBD antibodies induced by vaccination was 782,719 for subjects aged 18–55 years, 1,183,066 for subjects aged between 56 and 70 years and 3,638,522 for subjects over the age of 70 (12, 13). Apparently, older age groups did not show lower values of anti-RBD antibodies compared to younger groups. All these values were far superior to those recorded in HCS. After the second dose, the neutralizing activity of serum was assessed by different methods in all participants, obtaining values generally similar to those in the upper half of the distribution in HCS (12). Interestingly, a correlation was observed between the anti-RBD antibody titer and the neutralizing activity of the serum. Regarding the cellular T response, CD4^+^ T lymphocytes under stimulation of a pool of spike-specific peptides were strongly oriented toward the expression of T_H_1 cytokines (tumor necrosis factor α> interleukin-2> interferon γ), with a minimal production of T_H_2 cytokines (interleukin-4 and interleukin-13). In contrast, a low level CD8^+^ T lymphocytes response was detected only in the 100 μg dose group (12). In a later letter, data about immunogenicity 90 days after the second dose in 34 participants of the above studies, who received two injections of vaccine at a dose of 100 μg, were provided. Overall, despite a slight decline, both binding and NA values exceeded those observed among HCS in all age groups (14).

Recently, the results of the randomized and controlled phase III study (NCT04470427) have been published. The study assessed mRNA-1273 at a dose of 100 μg, administered twice 28 days apart, for the prevention of COVID-19 (26). A total of 30,420 participants were enrolled, of which 2.2% had a serologic or virologic data at enrolment compatible with previous COVID-19. Among participants who had received placebo, 185 cases of COVID-19 were documented, vs. 11 observed in participants who had received mRNA-1273, suggesting a vaccine efficacy of 94.1% (95% CI, 89.3–96.8). The efficacy of the vaccine was also confirmed in subgroups of participants such as those with previous COVID-19, or those older than 65 years.

## The Special Populations

A widely heterogenous group, immunocompromised patients can overall be regarded as a population of special interest for vaccine programs, as they may be at higher risk for COVID-19 than the general population (27) but, however, may not respond as well to the vaccine due to several alterations of the physiologic immune response (28). Data is not currently available to establish vaccine safety and efficacy of the approved vaccines in these groups.

### People Living With HIV

Despite data remaining limited, people living with HIV (PLHIV) infection were included in mRNA COVID-19 vaccine clinical trials. In the NCT04368728 trial (10), a total 121 PLHIV (0.3%) were included, 59 receiving BNT162b2 and 62 receiving placebo. In the NCT04470427 trial (23), a total 179 PLHIV (0.6%) were included at baseline, 87 in the mRNA-1273 arm and 92 in the placebo arm. In both instances, the studies do not report safety and efficacy data for this subset nor immunologic details.

### Solid Organ, Hematopoietic Stem-Cells Transplant Recipients and Malignancies

Recipient of solid organ, hematopoietic stem cell transplant and patients with active malignancies were excluded from the COVID-19 mRNA vaccine trials (10, 12). Therefore, there are no published data on the immunogenicity and interaction of mRNA-based antiviral vaccines with antineoplastic therapies. In the NCT04368728 trial, 733 patients in the BNT162b2 arm (3.9%) had a history of malignant disease, particularly 12 patients a history of leukemia (0.1%), 22 of lymphoma (0.1%), and 4 (0%) of metastatic solid tumor (10). Similar, patients with active oncological conditions have been excluded in the NCT04283461 trial. Thus, currently data on safety and efficacy of COVID-19 mRNA vaccines could only be inferred to these populations. Diverse approaches to mRNA-based vaccines, with therapeutic purpose, for patients with active malignancies have been employed in the last 10 years, without major concerns regarding safety (15, 29). On the other hand, data on the efficacy of mRNA-based COVID-19 vaccines lacks for immune-compromised hosts, a population with a background of suboptimal humoral and cellular responses to vaccinations (30).

### Individuals Receiving Immunosuppressing Drugs for Autoimmune Disorders

As for antineoplastic therapies, there is no data on the immunogenicity and interaction of mRNA-based antiviral vaccines with disease-modifying immunosuppressing drugs for autoimmune disorders. One-hundred eighteen patients with rheumatic diseases were included in the NCT04470427 trial (10), 62 receiving BNT162b2 (0.3%) and 56 receiving placebo (0.3%). No data on baseline treatments, safety and immunogenicity of mRNA vaccine is reported for this subcategory (10). Concerns regard the theoretical risk of disease flare after vaccination, in particular development of autoimmune syndromes related to type I interferon response stimulated by an mRNA/DNA vaccine (15), with specific concern of a SARS-CoV-2 antibody-mediated immune enhancement and a systemic inflammatory response.

## Vaccine-Enhanced Disease

Data from the study of SARS-CoV and other respiratory viruses suggests that anti-SARS-CoV-2 antibodies could exacerbate COVID-19, in the so called vaccine-enhanced disease (31), which can be linked to antibody-dependent enhancement (ADE) and vaccine-associated enhanced respiratory disease (VAERD) (32). ADE is an Fc–mediated enhancement of infection, with macrophage activation and cytokines secretion, typically associated with flaviviruses (33). VAERD is a distinct clinical syndrome, related to the production of an excessive amount of non-neutralizing antibodies, consequently to a T_H_2 biased immune response, which could potentially result in immune complex deposition and complement activation (32). Currently no evidence of ADE or VAERD occurrence among vaccinated individuals has been provided for SARS-CoV-2. Zhou et al. (34) showed in a convalescent individual with potent IgG neutralizing activity, that ADE is correlated to antibodies against specific non-overlapping epitopes of RBD. All the above-mentioned studies showed the production of relevant amount of NA, making VAERD unlikely. Moreover, data from mice (24) and humans (12), illustrated how immune response after vaccination is oriented toward a T_H_1 phenotype for both BNT162b1 (11) and mRNA-1273 (12).

## Discussion

Overall, the currently approved mRNA-based vaccines against COVID-19 displayed a reassuring safety and efficacy profile, with the latter above 94%. They can elicit specific antibodies titers and NA concentrations that are far superior from those observed among HCS, across a wide span of age. Moreover, the vaccine-induced T cellular response is oriented toward a T_H_1 response and no evidence of vaccine-enhance disease have been reported. Finally, initial evidence is showing, at least for BNT162b2, that vaccine efficacy is not hampered by mutations of SARS-CoV-2 spike protein, like those characterizing the UK and SA variants. These results are encouraging considering the enormous number of individuals receiving immunization. More data is eagerly awaited about the duration of specific and NA alongside with more detailed immunologic descriptions of humoral and cellular immunity, particularly in those with a previous COVID-19 infection or immunocompromised. Moreover, it is mandatory to quickly define the effectiveness of those vaccines against other viral variants and the role exerted in selecting them.

## Summary

Preliminary data shows how BNT162b2 and mRNA-1273 can elicit specific antibodies titers and neutralizing antibodies concentrations above those observed among COVID-19 human convalescent serum in the first 100 days after vaccination.

## Author Contributions

AL, AB, and AG conceived the study. AL wrote the first version of the manuscript. All the authors revised the final version of the manuscript.

## Conflict of Interest

The authors declare that the research was conducted in the absence of any commercial or financial relationships that could be construed as a potential conflict of interest.
